# Gene Amplification, ABC Transporters and Cytochrome P450s: Unraveling the Molecular Basis of Pyrethroid Resistance in the Dengue Vector, *Aedes aegypti*

**DOI:** 10.1371/journal.pntd.0001692

**Published:** 2012-06-12

**Authors:** Vassiliki Bariami, Christopher M. Jones, Rodolphe Poupardin, John Vontas, Hilary Ranson

**Affiliations:** 1 Department of Biology, University of Crete, Haraklio, Greece; 2 Vector Group, Liverpool School of Tropical Medicine, Liverpool, United Kingdom; Colorado State University, United States of America

## Abstract

**Background:**

Pyrethroid insecticides are widely utilized in dengue control. However, the major vector, *Aedes aegypti*, is becoming increasingly resistant to these insecticides and this is impacting on the efficacy of control measures. The near complete transcriptome of two pyrethroid resistant populations from the Caribbean was examined to explore the molecular basis of this resistance.

**Principal Findings:**

Two previously described target site mutations, 1016I and 1534C were detected in pyrethroid resistant populations from Grand Cayman and Cuba. In addition between two and five per cent of the *Ae. aegypti* transcriptome was differentially expressed in the resistant populations compared to a laboratory susceptible population. Approximately 20 per cent of the genes over-expressed in resistant mosquitoes were up-regulated in both Caribbean populations (107 genes). Genes with putative monooxygenase activity were significantly over represented in the up-regulated subset, including five CYP9 P450 genes. Quantitative PCR was used to confirm the higher transcript levels of multiple cytochrome P450 genes from the CYP9J family and an ATP binding cassette transporter. Over expression of two genes, *CYP9J26* and *ABCB4*, is due, at least in part, to gene amplification.

**Significance:**

These results, and those from other studies, strongly suggest that increases in the amount of the CYP9J cytochrome P450s are an important mechanism of pyrethroid resistance in *Ae. aegypti*. The genetic redundancy resulting from the expansion of this gene family makes it unlikely that a single gene or mutation responsible for pyrethroid resistance will be identified in this mosquito species. However, the results from this study do pave the way for the development of new pyrethroid synergists and improved resistance diagnostics. The role of copy number polymorphisms in detoxification and transporter genes in providing protection against insecticide exposure requires further investigation.

## Author Summary

Dengue is the most rapidly spreading arboviral infection of humans and each year there are 50–100 million cases of dengue fever. There is no vaccine or drug to prevent dengue infection so control of the mosquitoes that transmit this virus is the only option to reduce transmission. Removing mosquito habitats close to human homes can be effective but in reality most dengue control programmes rely on a small number of chemical insecticides. Therefore, when the mosquito vectors develop resistance to the available insecticides, dengue control is jeopardized. In this study we examined the causes of resistance to the insecticide class most commonly used in mosquito control, the pyrethroids. We found that a group of genes, which have been implicated in detoxifying these insecticides in other populations of dengue vectors, were highly over expressed in both these Caribbean populations and we investigated the molecular basis of this increased expression. The next steps, which will be a considerable challenge, are to utilize this information to develop effective means of restoring insecticide susceptibility in dengue vectors.

## Introduction

*Aedes* mosquitoes have shown a remarkable ability to develop resistance to insecticides [1]. Today, resistance to DDT, organophosphates and pyrethroids is widespread in the major dengue vector, *Aedes aegypti* [2] and this resistance is negatively impacting on control efforts. For example, in the Caribbean, resistance to pyrethroids is reducing the efficacy of pyrethroid space spraying in La Martinique and organophosphate resistance in Cuba is reducing the duration of control obtained by larviciding [3], [4]. Resistance to pyrethroids is of particular concern as this class of insecticides is increasingly replacing organophosphates in space spraying (WHO, 2011) and curtains, impregnated with pyrethroids, have also shown initial promise in reducing dengue transmission [5].

Most studies on the molecular basis of pyrethroid resistance focus on target site mutations [6], [7], [8]. Amino acid substitutions in the voltage gated sodium channel cause a resistance phenotype to pyrethroid insecticides known as knockdown resistance or *kdr*. At least four amino acid substitutions in the sodium channel (I1011M, V1016G, V1016I and F1534C) have been linked to resistance in *Ae. aegypti* [9], [10], [11], [12]. Two of these alleles, 1016I and 1534C, are widely distributed in the Caribbean [9]. The role of other resistance mechanisms is less clearly understood. Biochemical assays are frequently used to screen for metabolic resistance caused by elevated activities of cytochrome P450s, carboxylesterases and/or glutathione transferases. Although these assays lack sensitivity they have provided preliminary evidence that metabolic resistance is involved in conferring pyrethroid resistance in Caribbean populations of *Ae. aegypti* from Cuba, Grand Cayman, La Martinique and Trinidad .

To determine the molecular basis of this metabolic resistance and, identify other pathways potentially involved in conferring the resistance phenotype, we utilized microarray and quantitative PCR to analyse the near complete transcriptome of pyrethroid resistant populations from Cuba and Grand Cayman. The results confirm that elevated cytochrome P450 activity is strongly associated with pyrethroid resistance in these populations. Comparative analysis of the data from this, and earlier studies on populations from Latin America and Southeast Asia, indicates that the CYP9J family of P450 enzymes is primarily responsible for metabolic resistance to pyrethroids in *Ae. aegypti*.

## Materials and Methods

### Mosquito strains and sample collection

Three strains of *Ae. aegypti* were used in this study. The NEW ORLEANS (NO) strain is a laboratory strain that is susceptible to all known insecticides and was originally colonized by the Center for Disease Control and Prevention (CDC) Atlanta, USA. The pyrethroid resistant CAYMAN strain was colonized from larvae collected in routine field surveillance sites in Grand Cayman in 2008 . This strain has very high levels of resistance to DDT (>90% survival after 8 hours exposure to 4% DDT) and pyrethroids (resistance ratio of 109-fold to permethrin and 30-fold to deltamethrin compared with the susceptible New Orleans strain [9]). The CUBA-DELTA SAN 12 strain (CUBA-DELTA) was collected in 1997 in Santiago de Cuba. It was selected for 12 generations at the larval stage with deltamethrin at the Institute ‘Pedro Kouri’ in Havana, Cuba. CUBA-DELTA larvae were highly resistant to this insecticide (>1000-fold) and this resistance was also manifested at the adult stage [13].

Egg papers from the CAYMAN strain and the CUBA-DELTA strain were sent to the Liverpool School of Tropical Medicine, UK and the mosquitoes were reared under standard laboratory conditions (26°C, 80% RH) and a 12∶12 hours light∶dark cycle.

### Detection of target site mutations

The prevalence of the 1016I and 1534C *kdr* mutations in the CAYMAN strain has been reported previously. For the CUBA-DELTA strain, 38 mosquitoes were genotyped for the 1534C mutation using the tetraplex assay described in [9] and for the 1016I mutation using the hot oligonucleotide ligation assay (HOLA) [11].

### RNA extractions and labeling of cRNA

For each strain, total RNA was extracted from three pools of 30, three day old, non blood-fed females using Pico Pure™ RNA Isolation Kit (Applied biosystems, Foster city, CA, USA). The strains were reared in parallel to minimize variation resulting from breeding conditions. Each biological replicate consisted of mosquitoes from distinct generations to control for stochastic variations. The quality and concentration of RNA was assessed using a 2100 Bioanalyzer (Agilent technologies, Santa Clara, CA, USA). Then, 100 ng of total RNA were used for RNA amplification and labeled with Cy-3 and Cy-5 fluorescent dyes using the Two Colors Low Input Quick Amp Labeling Kit (Agilent technologies) according to manufacturer's instructions. Labeled cRNAs were purified with the Qiagen RNeasy spin columns (Qiagen, Hilden, Germany). Quantification and quality assessment of labeled cRNA were performed with the Nanodrop ND-1000 (Thermo Scientific, DE, USA) and the Agilent 2100 Bioanalyser (Agilent Technologies). Purified labeled cRNAs were stored at −80°C until microarray hybridizations.

### Hybridizations, data acquisition and statistical analysis

Hybridizations were made to the ‘Liverpool *Aedes aegypti* Agilent 8×15K v1’ microarray (A-MEXP-1966) designed by the Liverpool School of Tropical Medicine. Each array contains 60mer oligo-probes representing >14320 *Aedes aegypti* transcripts (93% of the putative gene count, 79% of putative transcripts –the lower coverage of transcripts is a consequence of the multiple putative transcripts for some genes). Labeled cRNA from CAYMAN and CUBA-DELTA were co-hybridized with age-matched NO samples, in direct pairwise comparisons. For two out of the three biological replicates, dye swaps were performed making a total of five hybridisations per comparison. Labeled targets were hybridized to the array for 17 h at 65°C and 10 rpm rotation and then washed according to Agilent protocol. Slides were scanned on Agilent G2565AA/G2565BA Microarray Scanner System using Agilent Feature extraction software (Agilent technologies). Genespring GX 11.1 software (Agilent technologies) was used for normalization and statistical analysis. To account for multiple testing , p-values were adjusted adopting the approach of Benjamini and Honchberg [14] to control for the false positives. Transcripts showing an absolute fold change >2-fold in either direction and a t-test P-value lower than P<0.01 after multiple testing correction were considered as significant. Descriptions and GO-terms of transcript-IDs were extracted from VectorBase [15] using BIOMART [16] and completed with Blast2GO software (BioBam Bioinformatics S.L. (Valencia, Spain)) [17]. GO term Enrichment analysis was performed on the significantly up-regulated genes (72% of transcripts present on microarray have GO-terms) using Blast2GO software with Fisher's exact test and false discovery rate (FDR)<0.05.

### Quantitative Real-time PCR

 Selected microarray data were validated using quantitative reverse transcription PCR (qRT-PCR). Primers were designed using the Oligo7 Primer Analysis Software (Molecular Biology Insights,Cascade, CO, USA) based on cDNA sequences retrieved from VectorBase. An aliquot of 4 µg total RNA from each of the three biological replicates, for each strain, served as a template for cDNA synthesis with Superscript III (Invitrogen, Carlsbad, CA, USA) using oligo-dT_20_, according to the manufacturer's instructions. The resulting cDNAs were diluted 20 times in ultra-high quality water for qRT-PCR reactions using a MiniOpticon System (Biorad, Hercules, CA, USA). PCR reactions of 25 µl contained Fast Start SYBR Green Master Mix (Roche, Penzberg, Germany), 0.3 µM of each primer (Table 1) and 5 µl of diluted cDNA. Melt curve analysis was performed to test the specificity of amplicons. A serial dilution of cDNA was used to generate standard curves for each gene in order to assess PCR efficiency and quantitative differences among samples. Primer sequences are provided in Table S1. The fold-change of each target gene, normalized to the 60S ribosomal protein L8 (AAEL000987) and 40S ribosomal protein S7 (AAEL009496), and relative to NO, was calculated according to the 2^−ΔΔCT^ method incorporating PCR efficiency [18]. In most cases, two independent primer sets were used for each gene (Table S1).

**Table 1:** Detoxification genes differentially expressed in pyrethroid resistant populations relative to the susceptible NO strain.

		Cayman vs NO		Cuba vs NO	
Transcript ID	Description	Fold change	Corrected p-value	Fold change	Corrected p-value
AAEL012836-RA	**cytochrome b561**	25.03	1.47E-03	20.43	2.37E-03
AAEL014617-RA	**cytochrome p450 CYP9J28**	14.21	9.08E-04	12.79	3.46E-03
AAEL014893-RA	**cytochrome p450 CYP6BB2**	8.45	4.00E-04	7.07	1.33E-03
AAEL014616-RA	**cytochrome p450 CYP9J27 v1**	8.41	5.84E-04	6.80	4.21E-04
AAEL003099-RA	**glucosyl glucuronosyl transferases**	6.99	7.50E-03	7.52	2.92E-03
AAEL014609-RA	**cytochrome p450 CYP9J26**	5.69	8.77E-04	4.86	5.86E-03
AAEL006717-RA	**abc transporter ABCB4**	5.13	6.25E-04	2.60	2.12E-03
AAEL006798-RA	**cytochrome p450 CYP9J10 v1**	4.17	6.89E-04	4.07	4.21E-04
AAEL000834-RB	**dimethylaniline monooxygenase**	3.68	2.09E-03	3.27	7.19E-03
AAEL012838-RA	**cytochrome b561**	3.11	1.13E-03	3.02	4.27E-03
AAEL007962-RA	**glutathione-s-transferase gste4**	2.87	7.26E-04	4.05	2.49E-03
AAEL006793-RA	**cytochrome p450 CYP9J9 v1**	2.51	1.14E-03	2.38	1.37E-03
AAEL014605-RA	**cytochrome p450 CYP9J9 v2**	2.48	7.98E-04	2.37	1.21E-03
AAEL014614-RA	**cytochrome p450 CYP9J10 v2**	2.25	3.22E-03	3.13	2.37E-03
AAEL014246-RA	**glucosyl glucuronosyl transferases**	2.17	8.37E-03	4.51	1.60E-03
AAEL003763-RA	**cytochrome p450 CYP329B1**	2.05	1.01E-03	2.18	2.75E-03
AAEL014611-RA	**cytochrome p450 CYP9J19 v2**	7.77	8.97E-04		
AAEL006810-RA	**cytochrome p450 CYP9J19 v1**	6.95	1.29E-03		
AAEL014607-RA	**cytochrome p450 CYP9J27 v2**	6.94	2.92E-04		
AAEL001288-RA	**cytochrome p450 CYP9M5**	3.25	2.18E-03		
AAEL009124-RA	**cytochrome p450 CYP6N12**	2.78	1.32E-03		
AAEL010500-RA	**glutathione-s-transferase gst GSTX2**	2.40	1.77E-03		
AAEL004086-RB	**aldo-keto reductase**	2.44	3.56E-03		
AAEL004086-RA	**aldo-keto reductase**	2.40	1.00E-03		
AAEL004724-RA	**carboxylesterase**	2.30	3.92E-03		
AAEL005113-RA	**alpha-esterase CCEae1A**	2.26	4.77E-03		
AAEL008138-RA	**abc transporter ABCG3**	2.19	5.11E-03		
AAEL005101-RA	**alpha-esterase CCEae4A**	2.19	2.24E-03		
AAEL009120-RA	**cytochrome p450 CYP6S3**	2.18	1.52E-03		
AAEL006044-RA	**cytochrome p450 CYP325Q1**	2.11	1.77E-03		
AAEL009129-RA	**cytochrome p450 CYP6Z9**	2.07	1.76E-03		
AAEL000545-RA	**carboxylesterase**	−2.73	4.19E-03		
AAEL000687-RA	**glucosyl glucuronosyl transferases**	−2.50	1.77E-03		
AAEL001804-RA	**glucosyl glucuronosyl transferases**	−2.38	3.00E-03		
AAEL003349-RA	**nadph-cytochrome p450 reductase**	−2.77	1.32E-03		
AAEL007473-RA	**cytochrome p450 CYP6AH1**	−7.37	6.46E-04		
AAEL009134-RA	**aldo-keto reductase**	−4.78	1.53E-03		
AAEL013096-RA	**aldo-keto reductase**	−2.79	2.91E-03		
AAEL014019-RA	**cytochrome p450 CYP4J16**	−2.40	6.99E-04		
AAEL014689-RA	**nadph cytochrome p450**	−3.19	1.04E-03		
AAEL015002-RA	**aldo-keto reductase**	−4.34	2.38E-03		
AAEL015475-RA	**cytochrome p450**	−2.18	3.99E-03		
AAEL002391-RA	**carboxylesterase CCEae5B**			14.09	8.47E-03
AAEL005937-RA	**atp-binding cassette transporter ABCC2**			3.53	9.85E-04
AAEL002046-RA	**cytochrome p450 CYP6CB1**			3.27	4.89E-03
AAEL002633-RA	**cytochrome p450 CYP9J31**			3.13	5.18E-03
AAEL002031-RA	**cytochrome p450 CYP12F7**			3.11	2.37E-03
AAEL002638-RA	**cytochrome p450 CYP9J6**			2.99	2.24E-03
AAEL009121-RA	**cytochrome p450 CYP6N9**			2.89	3.29E-03
AAEL014615-RA	**cytochrome p450 CYP9J23**			2.77	4.21E-04
AAEL009127-RA	**cytochrome p450 CYP6M11**			2.65	7.47E-04
AAEL014608-RA	**cytochrome p450 CYP9J8 v2**			2.56	7.35E-03
AAEL006811-RA	**cytochrome p450 CYP9J8 v1**			2.49	6.75E-03
AAEL008624-RA	**abc transporter**			2.43	1.88E-03
AAEL006802-RA	**cytochrome p450 CYP9J22**			2.30	1.43E-03
AAEL017297-RA	**cytochrome p450 CYP6M9**			2.29	4.08E-03
AAEL009117-RA	**cytochrome p450 CYP6M5**			2.29	5.18E-03
AAEL009131-RA	**cytochrome p450 CYP6Z8**			2.15	5.10E-03
AAEL002801-RA	**putative glutathione s-transferase**			2.10	4.62E-03
AAEL014612-RA	**cytochrome p450**			2.08	9.93E-03
AAEL014413-RA	**cytochrome p450 CYP304C1**			−2.09	9.03E-03
AAEL014891-RA	**cytochrome p450 CYP6P12 v2**			−2.67	5.21E-03
AAEL001078-RA	**glutathione s transferase GSTD1**			−3.34	5.20E-03

### Gene copy number analysis

Genomic DNA (gDNA) from three batches of ten adult mosquitoes from each strain was extracted using DNAzol (Invitrogen) according to the manufacturer's instructions. DNA quality and quantity was assessed by Nanodrop ND-1000 spectrophotometry and by running an aliquot on a 1.5% agarose gel. Quantitative PCR reactions were performed as described above on the same genes chosen for transcript analysis. The PCR efficiency for target and control genes (RPS7 and RPL8) was calculated from standard curves generated from a pool of gDNA for all three strains. 150–200 ng of gDNA was used as template and primer concentrations were between 100–300 nM for all genes. Primer sequences are provided in Table S1. The relative copy number fold-change was calculated using the 2^−ΔΔCt^ method [18].

## Results

### *kdr* genotyping

Two target site mutations, both previously associated with pyrethroid resistance in *Ae. aegypti*, are present at high frequencies in the two resistant strains from the Caribbean. In the CAYMAN strain, *kdr* frequencies of 0.79 for the 1016I allele, and 0.68 for the 1534C allele have been reported previously [9]. In the current study, 38 individuals from the CUBA-DELTA strain were genotyped and the frequency of the resistant alleles were 0.51 (1016I) and 0.88 (1534C).

### Microarray quality assessment and overall analysis

**Figure 1:**
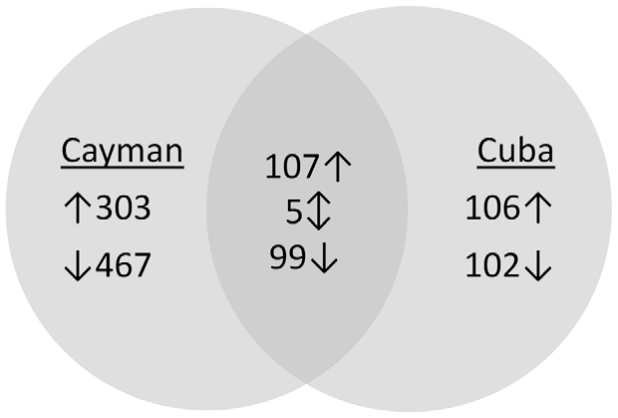
Summary of the genes differentially transcribed between resistant and susceptible strains. The Venn diagram shows the number of genes found significantly (P value<0.01) over- or under-transcribed (>2 fold in either direction) in one or both resistant strains compared to the susceptible New Orleans strain. Upward arrows indicate over- transcribed in resistant strains, downward represent under-transcribed.

Differences in gene expression in whole adult female mosquitoes of the pyrethroid-resistant strains from CUBA-DELTA and CAYMAN and the NO susceptible strain were assessed using a 15K *Ae. aegypti* microarray platform. The data have been deposited in ArrayExpress (accession number E-MTAB-868). Using an arbitrary cut off of fold change >2-fold in either direction and a t-test P-value lower than P<0.01 after multiple testing correction, 981 transcripts (5.4%) were differentially transcribed between CAYMAN and NO (410 up regulated and 566 down regulated) and 414 genes (2.2%) were differentially transcribed between the CUBA-DELTA and NO strains (213 up regulated and 201 down regulated) (Figure 1). Of the 516 up-regulated genes, 107 (20.7%) were over expressed in both resistant populations. In the down regulated subset, 99 of 668 (14.8%) genes were under expressed in both CAYMAN and CUBA-DELTA populations relative to NO. Five genes showed opposing patterns of gene expression between the two comparisons (Table S2).

The predicted functions of the genes differentially expressed in both populations were identified by BLAST2GO. More than 43% of the differentially expressed genes in the Cuba vs New Orleans comparison are annotated as ‘conserved hypothetical proteins’ in Vectorbase and 49% for Cayman vs New Orleans. These are listed in tables S2 to S4 but are not discussed further in this manuscript.

For further analysis we focused primarily on the subset of genes that were differentially expressed in both populations (Table 2), although other genes of interest are also discussed.

**Table 2:** Accession numbers and putative functions for the 20 genes showing the highest elevation in expression in the CAYMAN vs NO comparison and the 20 genes showing the highest decrease in expression in the same comparison.

		Cayman vs NO		Cuba vs NO	
Transcript ID	Description	Fold change	Corrected p-value	Fold change	Corrected p-value
AAEL009076-RA	nadh dehydrogenase subunit 4	72.55	0.00113	43.79	0.00890
AAEL012836-RA	**cytochrome b561**	25.03	0.00147	20.43	0.00237
AAEL015136-RA	niemann-pick type c-	21.31	0.00252	8.30	0.00635
AAEL002813-RA	coupling factor, putative	14.71	0.00069	9.74	0.00237
AAEL014617-RA	**cytochrome p450 CYP9J28**	14.21	0.00091	12.79	0.00346
AAEL000385-RA	developmentally regulated rna-binding protein	9.64	0.00455	4.62	0.00364
AAEL007083-RA	protein zer-1 homolog	9.16	0.00132	2.57	0.00464
AAEL014893-RA	**cytochrome p450 CYP6BB2**	8.45	0.00040	7.07	0.00133
AAEL014616-RA	**cytochrome p450 CYP9J27**	8.41	0.00058	6.80	0.00042
AAEL001668-RA	enolase	8.39	0.00098	10.78	0.00138
AAEL010227-RA	dolichol-phosphate mannosyltransferase	8.20	0.00143	10.93	0.00586
AAEL003099-RA	**glucosyl glucuronosyl transferases**	6.99	0.00750	7.52	0.00292
AAEL009798-RA	zinc finger protein	6.67	0.00160	4.03	0.00416
AAEL007849-RA	hypothetical protein AaeL_AAEL007849 [Aedes aegypti]	6.62	0.00164	5.28	0.00242
AAEL008390-RA	guanylate cyclase	6.50	0.00132	4.98	0.00496
AAEL004943-RA	riken cdna isoform cra_a	6.28	0.00149	8.51	0.00493
AAEL010761-RA	grip and coiled-coil domain-containing protein 1	6.09	0.00082	4.55	0.00042
AAEL001390-RA	hypothetical protein AaeL_AAEL001390 [Aedes aegypti]	6.08	0.00113	6.87	0.00153
AAEL014609-RA	**cytochrome p450 CYP9J26**	5.69	0.00088	4.86	0.00586
AAEL014645-RA	hypothetical conserved protein	5.59	0.00304	5.36	0.00237
AAEL012440-RA	sodium-bile acid cotransporter	−5.56	0.00393	−2.79	0.00320
AAEL003785-RA	uncharacterized protein kiaa0090	−5.57	0.00200	−2.23	0.00518
AAEL008025-RA	cg16787 cg16787-pa	−5.70	0.00029	−2.93	0.00320
AAEL011597-RA	pyridoxal phosphate phosphatase phospho2	−5.79	0.00108	−3.96	0.00990
AAEL009949-RA	homeotic antennapedia	−5.86	0.00051	−12.09	0.00252
AAEL006515-RA	bcdin3 domain containing	−6.76	0.00235	−8.02	0.00242
AAEL014128-RA	hypothetical protein AaeL_AAEL014128 [Aedes aegypti]	−8.04	0.00070	−3.91	0.00392
AAEL009462-RA	hydroxyacylglutathione hydrolase	−8.42	0.00049	−6.81	0.00042
AAEL009335-RA	adhesion regulating molecule 1 (110 kda cell membrane glycoprotein)	−8.60	0.00031	−3.18	0.00078
AAEL003485-RA	adhesion regulating molecule 1 (110 kda cell membrane glycoprotein)	−8.77	0.00031	−3.17	0.00129
AAEL013403-RA	hypothetical protein AaeL_AAEL013403 [Aedes aegypti]	−8.81	0.00070	−11.83	0.00042
AAEL005204-RA	isoform a	−10.21	0.00132	−7.68	0.00735
AAEL000219-RA	lactoylglutathione lyase	−11.40	0.00031	−5.47	0.00236
AAEL002812-RA	lyr motif-containing protein 5	−13.57	0.00164	−2.60	0.00501
AAEL000721-RA	deoxynucleotidyltransferase terminal-interacting	−13.99	0.00031	−12.24	0.00352
AAEL007244-RA	zinc finger protein	−16.33	0.00266	−2.46	0.00462
AAEL008079-RA	trypsin-	−17.70	0.00082	−6.14	0.00320
AAEL001336-RA	charged multivesicular body protein 2a	−36.40	0.00528	−3.42	0.00680
AAEL002811-RA	kda midgut protein	−77.13	0.00031	−3.39	0.00523

Detoxification genes are shown in bold.

### Genes differentially expressed in both populations

**Figure 2:**
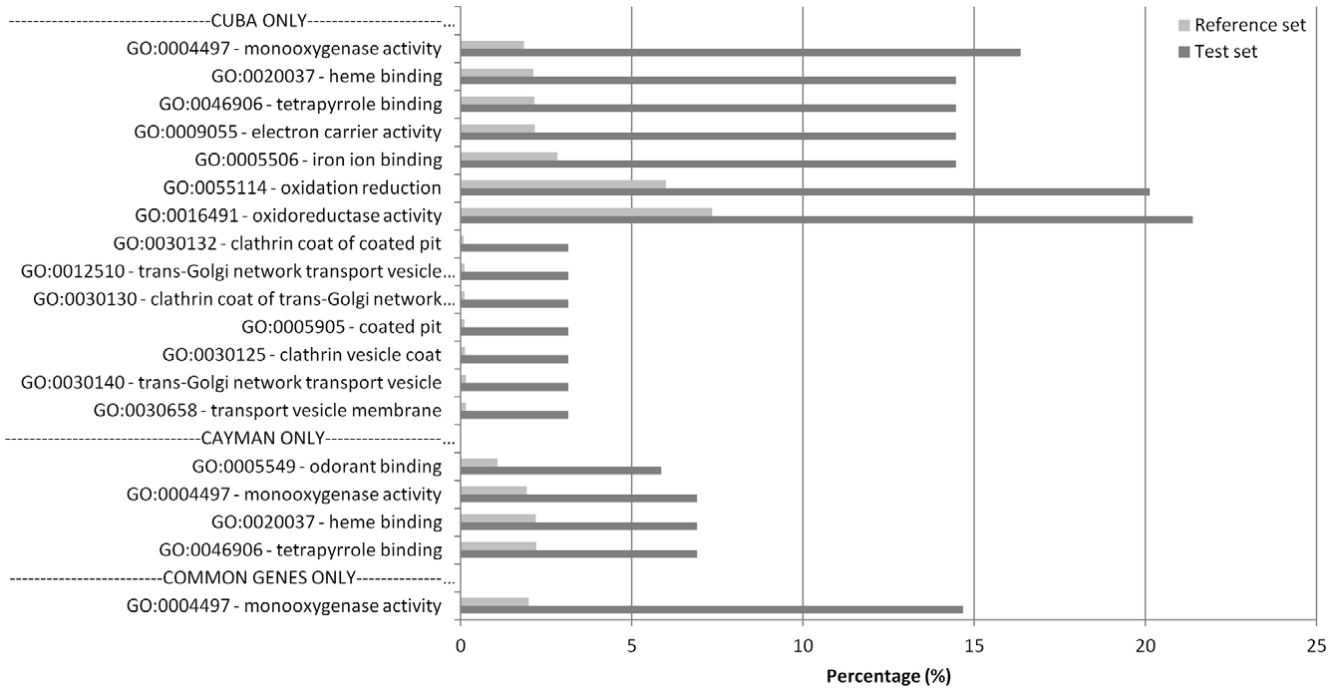
GO term enrichment analysis. The analysis was performed on the significantly up-regulated genes found in CAYMAN and CUBA-DELTA compared to NO. The BLAST2GO software was used for the annotation, mapping and enrichment analysis. The figure represents all the significant GO-term categories found significantly enriched compared to the reference set (all transcripts present on the microarray) after a Fisher's exact test and Benjamini and Hochberg multiple testing correction (Pval<0.05). The test set percentage indicates the percentage of up regulated genes belonging to a GO term category compared to all up-regulated genes used in the GO-term analysis while the reference set percentage indicates the percentage of a particular GO-term category compared to all genes with GO-terms on the microarray.

Enrichment analysis was used to identify particular GO terms that were over represented in the subset of transcripts up regulated in both resistant populations. Thirteen GO terms were significantly over represented in the up-regulated subset (Figure 2). However, after Benjamini and Hochberg multiple testing correction (Pval<0.05) only the GO term designating moooxygenase activity was significantly differentially represented.

This initial screening via GO terms was followed up with a manual examination of the putative functions of each of the 107 transcripts up-regulated and 99- down regulated in each population. This gene set, ranked by fold change in the CAYMAN population, is listed in Table S2. Genes with putative detoxification functions are listed in Table 1. Detoxification genes comprised 15.8% of the commonly up-regulated subset but were not represented at all in the down –regulated subset of the commonly expressed genes. Several additional detoxification genes were found up-regulated in either the Cayman or Cuba strains (Table 1).

### Detoxification genes

A total of 18 and 13 *CYPs* are over expressed >2-fold in the CUBA-DELTA strain and CAYMAN strain relative to the susceptible NO population respectively (Table 1). Seven of these *CYPs* were up-regulated in both strains: *CYP6BB2*, *CYP9J9*, *CYP9J10*, *CYP9J26*, *CYP9J27*, *CYP9J28* and *CYP329B1*. Three P450s were down regulated in the CAYMAN strain and two in the CUBA-DELTA strain but none of these are common to both strains. Twelve of the 24 up-regulated *CYPs* (and five of the seven up-regulated in both strains) belong to the CYP9J family.

Further genes with roles in oxidative metabolism of xenobiotics were amongst the subset of genes over expressed in both resistant populations. This included a dimethylanaline monooxygenase (AAEL00834), a member of the cytochrome b561 family (AAEL012836) and subunit 4 of NADH dehydrogenase (AAEL009076). Other detoxification genes included the glutathione transferase, GSTe4 (AAEL007962), and two glucosyl glucornosyl transferases (AAEL003099 and AAEL014246) (Table 1).

Seven P450s (plus an ABC gene described below) were selected to validate the microarray results by qPCR. These included six out of the seven P450s over expressed in both strains and an additional P450 that is only over expressed in the CAYMAN strain, *CYP9J19*. In general there is good agreement between the qPCR and microarray data (Table S5) with the exception of the *CYP6BB2* gene, (AAEL014893). The high level of over expression of this gene observed in the microarray could not be confirmed by qPCR using two alternative primer sets. However, the qPCR confirmation of over expression of the CYP9J genes adds further support for these enzymes playing a role in resistance to pyrethroids in these Caribbean populations.

### Other genes of interest

**Figure 3:**
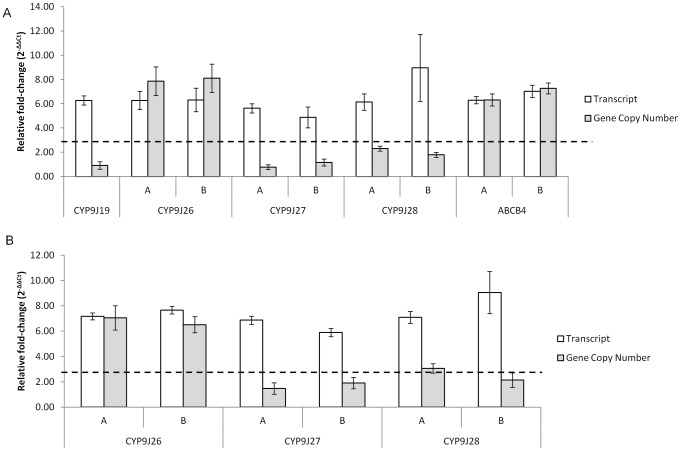
Quantitative PCR analysis of selected genes from the microarray experiments. Relative-fold change in transcript and gene copy number normalised to two ribosomal genes was compared between the resistant CAYMAN (A) and CUBA-DELTA (B) strains against the NEW ORLEANS susceptible strain. Transcript levels are shown by the white columns and gene copy numbers by the grey columns. Error bars represent 95% confidence intervals.

An ABC transporter gene, AAEL006717, was expressed at approximately 5-fold and 2-fold higher levels in the pyrethroid resistant populations from Cayman and Cuba respectively, relative to the susceptible NO strain. This gene is potentially of interest because elevated ABC transporters have been linked to insecticide resistance in several species [20], [21], [22], [23] although the physiological mechanism by which these transporter proteins act to reduce insecticide susceptibility is unknown. The over expression of AAEL006717, which is an orthologue of the *An. gambiae ABCB4* gene, [24] was confirmed by qPCR for the CAYMAN strain but not attempted in the Cuban population (Figure 3 Table S5).

In the CAYMAN strain, 8 transcripts for odorant binding proteins (OBPs) were up-regulated. Furthermore, when the CAYMAN population was analysed alone, the GO term ‘odorant binding’ was the most differentially represented term in the up-regulated set of transcripts (Figure 2). OBPs facilitate the passage of semio-chemicals across the antennae, and other sensory appendages, to the olfactory neurones. To date, no specific role for OBPs in insecticide resistance has been demonstrated but this is not the first time that OBPs have been identified as being over expressed in insecticide resistant populations. A study of bendiocarb resistance in *Anopheles* identified an OBP gene that was overexpressed in Ghanaian resistant populations (S Mitchell, unpublished data).

### Gene amplification associated with elevated levels of ABC transporter and P450 transcripts

Quantitative PCR was used to compare gene copy number between the two resistant and the susceptible *Ae. aegypti* strains. Using the same cut off of >2 fold change in expression, gene amplification was observed for two genes, *CYP9J26* and the ABC transporter, *ABCB4* (AAEL006717). The copy number of *CYP9J26*, measured using two different primer pairs, was between 6.5 and 8.1-fold higher in the resistant CUBA and CAYMAN strains respectively compared with New Orleans (Figure 3). Similarly, the *ABCB4* gene was amplified approximately 7-fold in the Cayman strain relative to the New Orleans (Figure 3).

## Discussion

Pyrethroid resistance is widely distributed in *Ae. aegypti* throughout its range but relatively little is known about the mechanisms responsible for this resistance. Target site resistance is present in both the Cuban and Cayman populations although neither of the two mutations, 1016I or 1534C, were fixed in either population, despite several rounds of laboratory selection with deltamethrin in the Cuban strain. Interestingly the 1534C mutation has recently been shown to confer a selective advantage against type I pyrethroids but not affect the sensitivity to type II pyrethroids such as deltamethrin [25]. As far as we are aware, electrophysiological experiments have not been performed to examine the impact of the V1016I substitution. Thus, target site resistance may be partially responsible for the high levels of deltamethrin resistance in both these populations but it is likely that other mechanisms are involved.

In this study, a microarray containing probes for the vast majority of annotated genes in the *Ae. aegypti* genome was used to compare gene expression in the two Caribbean populations with a standard lab susceptible strain. A potential limitation of this approach is the use of a single laboratory susceptible strain that originated from the United States. Ideally, a range of susceptible strains including wild populations from similar genetic regions would be included in the study. Unfortunately, such strains are becoming increasingly difficult to find. The differential gene expression observed may be partially attributed to the different genetic background of the strains although, encouragingly, earlier experiments have shown no significant difference in expression of detoxification genes between New Orleans and another well established laboratory susceptible strain, Rockefeller [19]. In light of this potential criticism, the analysis focused primarily on genes that were up-regulated in both resistant populations. Interestingly, a smaller number of genes were found differentially transcribed in the Cuban strain, which had been subject to extensive laboratory selection, than the Cayman strain, which was resistant upon colonization. By using GO term enrichment analysis, the functions or processes that were enriched in the subsets of genes up or down regulated in the pyrethroid resistant populations were identified. Only one GO term was significantly enriched in this analysis. Eleven transcripts with the GO term GO:0004497, monooxygenase activity, were found amongst the subset over expressed in both insecticide resistant populations. This supports the well documented role of cytochrome P450s in conferring pyrethroid resistance [26].

*Aedes aegypti* has an extensive repertoire of between 160 and 180 *CYP* genes [19]. The uncertainty over the exact gene count is partly due to the fragmented nature of the *Ae. aegypti* genome assembly; several supercontigs containing clusters of P450s most likely represent alternative haplotypes. P450s contained within these putative duplicate clusters have been assigned independent accession numbers in VectorBase but named as allelic variants of the same P450 by the P450 nomenclature committee (designated v1 or v2). This issue is discussed further in the supplementary material of Strode et al (2008) [19]. For ease of discussion, in the current manuscript, official P450 nomenclature has been used to discuss the P450 family and the v1/v2 nomenclature omitted. However, the finding that two variants of the same gene frequently show similar fold changes in expression, adds confidence to the analysis as the probes were not designed to be able to distinguish allelic variants.

**Figure 4:**
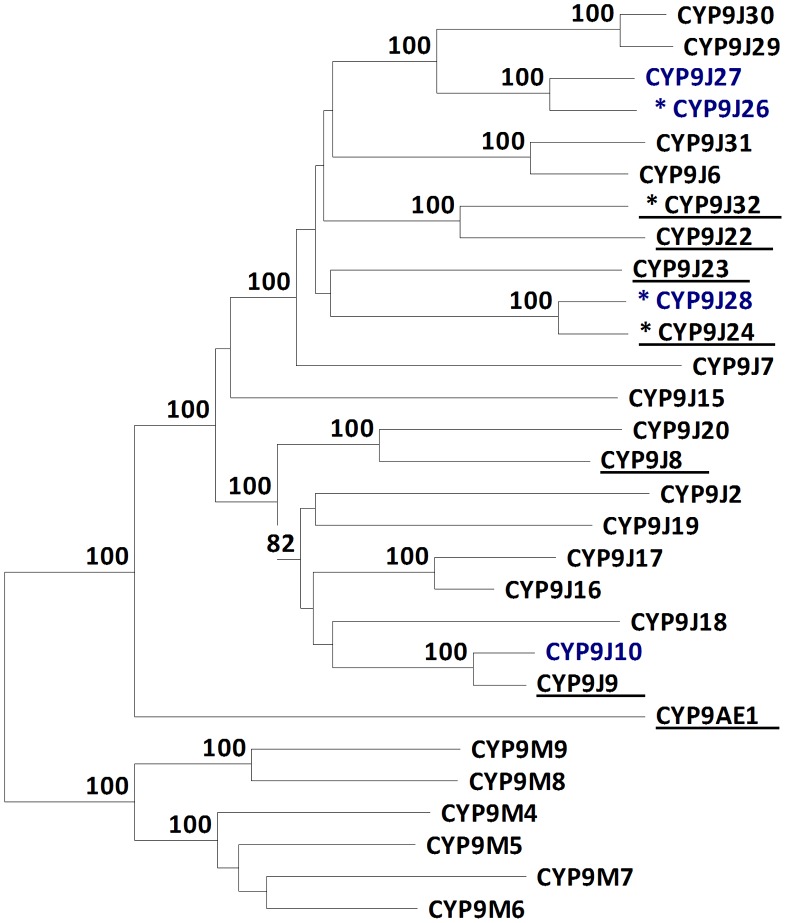
Unrooted distance neighbour joining tree showing phylogeny of *Aedes aegypti* CYP9 genes. Nodes with >70% bootstrap support (500 pseudoreplicates) are indicated. Sequences in blue are up-regulated in both CUBA-DELTA and CAYMAN strains from the current study. Sequences underlined are over expressed in ≥2 populations versus laboratory susceptible strains from previously published studies (see text for further details). Sequences marked with * have proven ability to metabolise pyrethroids (Stevenson et al, submitted).

Seven cytochrome P450 genes were up-regulated in both resistant populations. This included two genes in the CYP6 clade, *CYP6BB2* and *CYP329B1*, but neither was confirmed by qPCR. The five remaining P450 genes belonged to the CYP9J family. Four of these have been found to be up-regulated in resistant strains from other geographical localities. *CYP9J9* and *CYP9J10* were found over expressed in Thai and Latin American populations of *Ae. aegypti*. *CYP9J28* is over expressed in pyrethroid resistant populations from Peru and Mexico [19], [27], and has also been shown to be over expressed in pyrethroid resistant *Ae. aegypti* from Vietnam (Warr and Ranson, unpublished data). *CYP9J27* is over expressed in Thailand [19] and is also one of the candidates emerging from the Vietnam study. Other CYP9J genes have also been implicated in resistance (Figure 4). In fact, in total, ten CYP9J genes have been found over expressed in at least two pyrethroid resistant populations. Four of these, *CYP9J24, 26, 28* and *32*, have now been biochemically characterized [28] and have all been shown to metabolize pyrethroids (a single CYP6, CYP6CB1 was also expressed but had no activity against this insecticide class).

Aside from the CYP9s, the only additional clade of *Ae. aegypti* P450s that is found repeatedly over expressed in resistant strains is the CYP6Z subfamily. Genes *CYP6Z6*, *Z8* and *Z9* have been found over expressed in multiple populations from southeast Asia, Latin America and Caribbean [19], [27], [29] and *CYP6Z8* and *Z9* were over expressed in Cuba and Cayman populations respectively in the current study.

The CYP9 family in *Ae. aegypti* is greatly expanded compared to other insect species with over three times as many members as found in *An gambiae* and nearly six times as many as in *D. melanogaster* [19], [30]. The degree of genetic redundancy in the P450 family of *Ae. aegypti* makes it unlikely that a single gene responsible for pyrethroid resistance in all strains will be detected, particularly if resistance is emerging independently in different populations. However, the identification of a small subset of genes, consistently over expressed in resistant populations does suggest it should be possible to develop specific inhibitors of these metabolic pathways that could be used as insecticide synergists.

Gene amplification was associated with the over expression of one of the P450 genes, *CYP9J26*, in both strains, with approximately 7 –fold increase in copy number compared to the susceptible strain. Increased gene copy numbers have been associated with P450 mediated resistance in *An. funestus*, *D. melanogaster* and *Myzus persicae* [31], [32], [33]. The increased transcript levels of the other CYP9 genes were not associated with an increase in gene copy number although copy number polymorphisms appear to be common in the P450 family in *Ae. aegypti* (Strode et al, 2008). *CYP9J26*, *27* and *28* are arranged sequentially in the *Ae. aegypti* genome within a large cluster of CYP9 genes on supercontig 1.1188. It is not yet known if the *CYP926* duplications are found in tandem. Multiple copies of the ABC transporter were also present in the Cayman resistant population. Gene amplification is being increasingly recognized as an important mechanism conferring metabolic resistance to insecticides with examples reported in all the major families of detoxification enzymes from several insect species [34].

Resistance to pyrethroid insecticides is now widely established in *Ae aegypti* populations throughout its distribution [2]. Elucidating the mechanisms responsible for this resistance will facilitate resistance monitoring and pave the way for the development of effective resistance reversal approaches. The microarray approach used in this study is not itself a field applicable screening approach. However, using this tool to analyse additional pyrethroid resistant populations will help define a subset of genes that are responsible for pyrethroid resistance. As discussed above, although it is unlikely that a single diagnostic mutation will be detected, the identification of a panel of candidate resistance associated genes is an important prerequisite for developing simple, molecular diagnostics that are urgently needed by dengue control programmes. Given the key role that pyrethroids play in controlling this disease vector, and the lack of affordable, acceptable alternative insecticides, it is imperative that efforts are made to monitor for resistance and reduce the impact that this resistance may impose on vector control interventions.

## Supporting Information

Table S1 **Primer sequences used for quantitative PCR.**
(DOCX)

Table S2 **List of genes differentially expressed in both the CAYMAN and CUBA-DELTA strain compared to New Orleans.**
(XLS)

Table S3 **List of genes differentially expressed in the CAYMAN strain compared to New Orleans.**
(XLS)

Table S4 **List of genes differentially expressed in the CUBA-DELTA strain compared to New Orleans.**
(XLS)

Table S5 **Differential expression of genes determined in quantitative PCR experiments.** Data analysis was performed according to the ΔΔ_CT_ method taking into account PCR efficiency [35] and normalized with two control genes 60S ribosomal protein L8 (AAEL000987) and the 40S ribosomal protein S7 (AAEL009496). Each experiment was analyzed separately. Results were expressed as mean transcription ratios ± SD (n = 3).
(XLS)
